# Trends and challenges in modeling glioma using 3D human brain organoids

**DOI:** 10.1038/s41418-020-00679-7

**Published:** 2020-12-01

**Authors:** Aruljothi Mariappan, Gladiola Goranci-Buzhala, Lucia Ricci-Vitiani, Roberto Pallini, Jay Gopalakrishnan

**Affiliations:** 1 Institute of Human Genetics, University Hospital Düsseldorf, Heinrich-Heine-Universität, Universitätsstr. 1, 40225 Düsseldorf, Germany; 2 Department of Oncology and Molecular Medicine, Istituto Superiore di Sanità, Viale Regina Elena 299, 00161 Rome, Italy; 3 Depatment of Neuroscience, Fondazione Policlinico Universitario A. Gemelli, Largo Agostino Gemelli 8, 00168 Rome, Italy

**Keywords:** Cancer stem cells, CNS cancer

## Abstract

The human brain organoids derived from pluripotent cells are a new class of three-dimensional tissue systems that recapitulates several neural epithelial aspects. Brain organoids have already helped efficient modeling of crucial elements of brain development and disorders. Brain organoids’ suitability in modeling glioma has started to emerge, offering another usefulness of brain organoids in disease modeling. Although the current state-of-the organoids mostly reflect the immature state of the brain, with their vast cell diversity, human brain-like cytoarchitecture, feasibility in culturing, handling, imaging, and tractability can offer enormous potential in reflecting the glioma invasion, integration, and interaction with different neuronal cell types. Here, we summarize the current trend of employing brain organoids in glioma modeling and discuss the immediate challenges. Solving them might lay a foundation for using brain organoids as a pre-clinical 3D substrate to dissect the glioma invasion mechanisms in detail.

## Introduction

Glioma is an aggressive form of brain cancer characterized by poor prognosis [1, 2]. Despite surgical resection, glioma patients succumb due to its rapid growth in the brain, resistance to chemotherapy, and high invasiveness with a median survival time of ~15 months, and there is no cure. As a result, the 5-year survival rate remains less than 10% [3–5]. Gliomas possess glioma cancer stem cells (GSCs) that underlie tumor initiation, progression, and recurrence after initial treatment. Furthermore, GSCs exhibit characteristics of neural stem cells such as expression of neural stem cell markers and self-renewability. Thus, characterization of GSCs has been critically useful to understand the fundamental biology of glioma [6–10].

GSCs growing in adherent 2D cultures allow studying specific aspects of tumor biology. Furthermore, these 2D GSCs cultures offer possibilities to conduct chemical and genetic screens, transcriptomics, proteomics, clonal or single-cell analyses without the influence of extrinsic signals. Interestingly, early-passaged GSCs retain the genetic and transcriptional signature of the parental tumor, and thus low-passaged GSCs are widely used as an accepted model of glioma [11]. However, studying GSCs in 2D is a particular challenge due to spontaneous changes in stem cell properties and a high degree of stochastic intercellular variations, ultimately resulting in cell populations markedly different from the original tumor. Besides, the invasive behavior of GSCs cannot be modeled in 2D culture, as it requires 3D like brain-like tissue as a host. Therefore, studies have focused on isolating GSCs from primary tumor biopsies and subsequently used them to transplant in mouse models (Patient-derived Xenografts, PDXs). These studies have revealed the surprising plasticity of GSCs reflecting the in vivo tumor heterogeneity [12–14]. Thus, GSCs, when grown in 2D, tend to lose their intrinsic heterogeneity due to the lack of spatial interactions with neighboring cells, extracellular matrix, and tumor microenvironment. As a result, drug-screening studies purely relying on 2D cultures have their downside, as the identified agents may not be therapeutically active in clinical studies.

Last decade researches have seen impressive progress in understanding the fundamental aspects of glioma biology in terms of genetic factors that can elevate intra-tumor heterogeneity, differential behaviors of GSCs, and changes in their growth and invasion behaviors [15–18]. Nevertheless, these efforts have not offered a significant benefit to patients. This could be due to the lack of physiologically relevant a pre-clinical human in vitro system that can reliably recapitulate GSCs invasion behaviors and suitable to adopt robust drug screening studies. Undeniably, a more sophisticated experimental system mirroring the tumor environment, yet allowing experimental manipulations, is required to predict the GSCs behavior accurately. Indeed, recent advances in 3D culturing methods have resulted in so-called “organoids”, a 3D structure constituting various cell types allowing their spatio-temporal interactions. On the other hand, when GSCs assemble into glioblastoma organoids (GBOs), they could recapitulate in vivo-like heterogeneity [19, 20]. While these GBOs can exhibit in vivo-like features, they do not offer the possibility to study the invasion behavior of GSCs in the human brain tissue. While traditional patient-derived xenografts (PDXs) in mice allowed addressing crucial insights into GSCs invasion behavior [21, 22], they lack the human tumor microenvironment. In addition, they fall short because of their lengthy process and incompatible with being applied for drug screening studies [23]. Thus, the lack of suitable in vitro models that mirror the complexities of the human brain is a limiting factor in understanding the fundamental invasion behavior of GSCs in the human brain. In this review, we discuss the applicability of recently emerged 3D human brain organoids in modeling glioma and discussing future perspectives of using them as a pre-clinical model to predict the clinical outcome and use them as a personalized drug-screening platform.

## The emergence of human brain organoids and their applications

Brain organoids are an innovative experimental model of modern reductionists’ approach. It is noteworthy that the early developmental neurobiologists in the last century have already adapted similar deconstructive strategies to deconstruct the complexities of human brain development [24, 25]. In the past, researchers did not have access to currently available pure cultures of pluripotent cells, but their approaches relied on ex vivo tissues that already harbored resident stem cells. Thus, with the combination of now available embryonic stem cells (ES) or induced pluripotent cells (iPSCs), modern 3D culturing technologies and directed differentiation protocols have enabled us to exploit the self-organization ability of pluripotent stem cells to generate human brain-like tissues named brain organoids. These 3D tissues recapitulate many aspects of neural epithelial cells cytoarchitecturally similar to the developing human brain. The historical aspects of different experiments that led to the generation of modern-day brain organoids are described elsewhere [26, 27].

In this modern era of brain organoids, two laboratories stand out to receive the full credit for their pioneering effort in generating the first kind of 3D neural tissues in vitro. Attempts by the Sasai and Vaccarino laboratories have generated self-organized structures from pluripotent cells that recapitulated several neuroretina and telencephalic development [28–30]. These neural tissues stunningly displayed a brain-like architecture constituting polarized radial glia, intermediate progenitors, and layer-specific cortical neurons similar to their in vivo tissues’ cytoarchitecture. Their seminal works have defined self-assembly principles and laid the foundations for the number of laboratories to generate so-called “3D human brain organoids” [31, 32]. Technological developments by other labs have then brought brain organoids into the limelight, which helped others decoding the early events of brain development, including interneuron migration and cell diversity analysis [33–35]. In this context, the Gopalakrishnan lab has utilized early brain organoids to dissect microcephaly mechanisms due to genetic mutation and identified shared mechanisms of microcephaly due to genetic mutation and Zika virus infections [36–38]. The Guo Li-Ming lab has devised a method to generate region-specific brain organoids that have further refined target cell types of Zika virus [39]. The Arlotta lab revealed the extensive cell diversities of human brain organoids [35]. Lately, brain organoids have also surprisingly helped to model neurological COVID-19 [40]. Several elegant works have further shined the original brain organoid generation protocols, which we apologize for not mentioning here due to lack of space. Many review works have comprehensively listed various kinds of brain organoids, methods, and their use ranging from classical developmental biology to modeling pathomechanisms of multiple diseases, including glioma [27, 41–44]

## Do human brain organoids suitable 3D host tissues for GSCs?

Unlike other cancer types, gliomas metastasize rarely but infiltrates diffusively into the brain [45, 46]. Thus, despite the region-specific resection of glioma tumors, the diffused glioma spreading into the rest of the brain tissue is persistent. Clinically, this is challenging and is a significant bottleneck in therapeutics. 2D cultures do not help to assay such an invasive behavior of GSCs. Therefore; it became essential to model the diffusiveness of GSCs in brain mimetics using 3D brain organoids as it may provide the first glimpse of GSCs’ behavior in the host tissues. Imaging at high resolution, the infiltration assay may map the migratory characteristics of GSCs in the human brain. Finally, one can optimize and upscale the assay that can be extended as personalized glioma invasion and drug screening assays. As described before, only a handful of studies have just started to recognize brain organoids’ power to study the glioma invasion mechanisms.

Bian and colleagues explored the use of genetically engineered brain organoids and expressed oncogenes ectopically to develop tumors. The observations of ectopic growth of tumor-like mass in organoids did not sufficiently help assessing to what extent those cells’ invasion behaviors mirror the biological relevance [47]. More recently, Ballabio et al. used human cerebellar organoids to understand the molecular mechanisms of medulloblastoma development and identified that Otx2 and c-MYC as inducers of medulloblastoma [48]. Interestingly, the authors have extended their system to assay the anti-tumor activity of Tazemetostat, an EZH2-specific inhibitor that could antagonize Otx2/c-MYC-mediated tumorigenesis. Their study identifies the usefulness of brain organoids in identifying deregulated pathways of tumor formation. Ogawa et al., on the other hand, made a further refinement in the assay by implanting patient-derived glioma cells and provided an insight that the foreign materials can establish tumor-like structures in the human brain organoids [49]. The Fine lab then used human embryonic stem cell (hESC)-derived brain organoids to host clinical glioma samples and showed that the behaviors of GSCs such as tissue invasion and tumor formations, phenocopying patient gliomas. Their work also provided the first glimpses of GSCs-penetrating organoids for chemo and radiotherapy, thereby setting how one can exploit the brain organoids for glioma modeling [50]. In summary, both of these initial works have highlighted an important point that GSCs have preferential tropism to organoid tissues even though brain organoids are far from the human brain tissue in terms of mature cell types and vasculatures.

To further optimize the GSCs invasion assay, Goranci-Buzhala et al. tested various parameters such as organoid’s age, GSC numbers, and the order of GSCs addition either single cells or spheres [51]. Their attempts have devised a rapid and efficient glioma modeling. Importantly, their assays can distinguish different invasion behaviors of primary and recurrent patient derived GSCs. Tissue clearing and high-resolution quantitative 3D imaging have revealed unbiased identification and quantifications of invasion protrusions, depth of invasion, and volume occupied within the host tissues of brain organoids. Strikingly, these assays have shown a surprising finding that GSCs invasion’s propensity was inversely co-related to organoids age, indicating that mature neuronal types can provide a favoring microenvironment for the GSCs. Earlier studies have shown that neuronal activities release mitogenic factors that can support glioma cell proliferation in the host brain tissues [52]. Trying to mimic this aspect, Goranci-Buzhala et al. took an orthogonal approach by providing synaptic protein Neuroligin-3 (NLGN-3) exogenously. They identified that indeed NLGN-3 could enhance the invasion behavior of GSCs in early-stage organoids, which are predominantly at the stage of active proliferation [51]. All of these findings provide surprising hints that brain organoids can provide an ex-vivo-like experimental system enabling to map the invasion behavior of GSCs quantitatively.

**Fig. 1:**
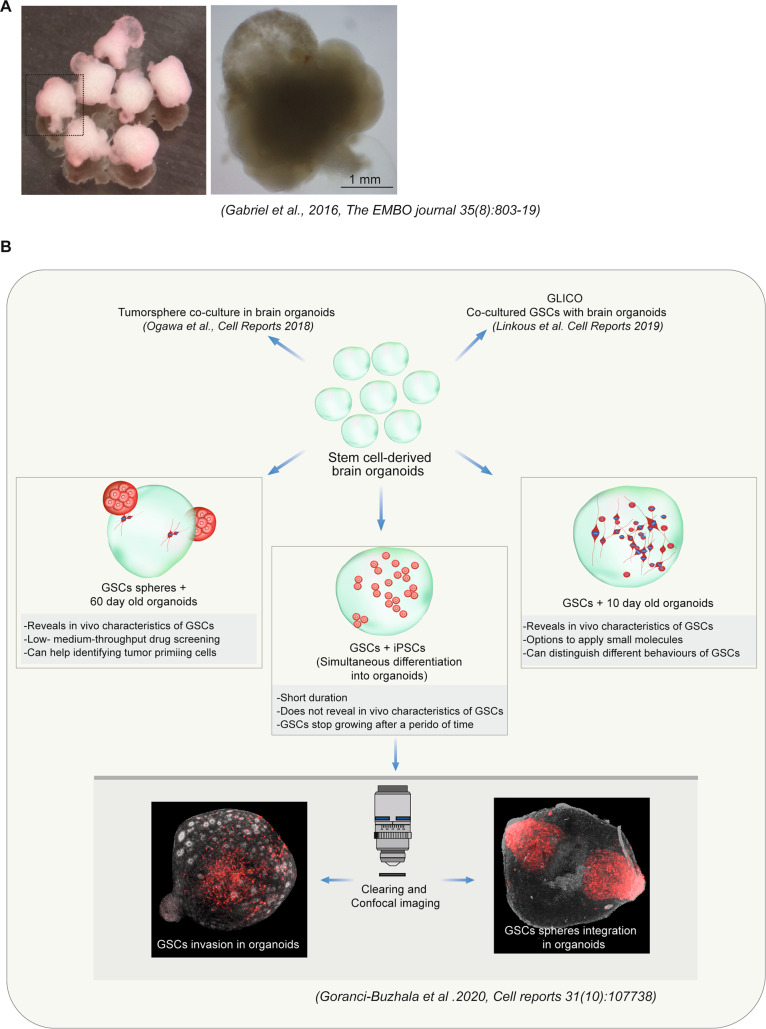
Examples of iPSCs-derived human brain organoids. **A** Brain organoids shown here exhibits morphologically similar appearances of human neural epithelial tissues with fluid-filled regions. The images adopted from Gabriel et al. EMBO Journal 2016. **B** Schematic illustrations of various glioma invasion assays that used patient derived GSCs and human brain organoids. Schemes also explain different methods of optimization and what they can reveal.

Based on the observations, it is intuitive that GSCs not only invade into the brain but also interact with the neuronal cells to integrate and to exert its effect to disrupt normal brain functions. To test this aspect, Goranci-Buzhala, and colleagues cultured organoid slices that could generate long-range axons growing as neuronal outgrowths [51]. Importantly, these neurons represented mature neuronal cell types [36]. GSCs surprisingly interacted with neurons establishing hemisynapses-like structures [51]. Strikingly, similar GSCs-neuronal interactions have also been recently described in mouse xenografts [53]. It remains to be tested whether the integrated GSCs human brain organoids exhibit electric activities. Figure 1 schematically summarizes the current state of glioma invasion assays in human brain organoids.

In summary, even though brain organoid-based glioma invasion assays still at the level of development, the findings so far are encouraging that brain organoid tissues can serve as a 3D substrate for clinical GSCs. Nevertheless, the field requires significant refinements, which we outline in the following section. Table 1 summarizes the currently available 3D culture systems to study glioma invasion biology.

**Table 1:** Summary of 3D cultures used in glioma research.

Model	Advantages	Limitations	References
GSCs as 3D floating neurospheres	-Genetic and transcriptional state of parental tumor can be retained -Polarization and 3D organizations can be maintained	-Interaction with brain tumor microenvironment is lost	[12, 65]
3D glioma organoids (GBO) in matrigel	-Modeling of necrotic/hypoxic human tumor -Modeling of quiescence/proliferation and differentiation -Rapid growth and long term culturing -Gradient of stem cells (hypoxia, Sox2+) -Growth of different CSCs cellular hierarchies	-Does not provide GSCs invasion behaviors as it lacks the host tissue	[20]
3D GBOs in serum free conditions without EGF and bFGF2	-Rapid and reproducible protocol (2 weeks) -Cell selection is avoided -Inter-tumoral and intra-tumoral heterogeneity preserved -Hypoxic gradient -Cryopreservation -Genetic and molecular signatures maintained -Applicable to drug sensitivity tests	-Does not provide GSCs invasion behaviors as it lacks the host tissue	[19]
Human brain organoids (NeoCor) genetically engineered by CRISPR/Cas9	-To study the mechanisms of glioma-like tumor progression in human brain-like tissues	-Does not recapitulate the genomic complexity -Does not reflect subtypes -Does not resemble to patient-derived GSCs	[47] [49]
Co-culturing of GSCs in human brain organoids	-Rapid assay to analyze the growth behavior of GSCs in brain-like tissues	-Does not recapitulate GSCs invasion behavior -Does not reveal interaction mechanisms of GSCs to the host cell types	[49]
Co-culture of GSCs with 3D brain organoids (GLICO, GLIoma Cerebral Organoids)	-Allows extensive invasion assays -Heterogeneity in growth and invasion behavior similar to the original tumor	-Absence of vasculatures and immune cells -Does not reveal interaction mechanisms of GSCs to the host cell types	[50]
Co-culture of patient-derived cancer cells and vascular endothelial cells in de-cellularized brain extracellular matrix (3D bioprinting technologies)	-Oxygen gradient is maintained -Perivascular niche reproduced -Spatial heterogeneity of stem cell types are maintained -Prediction of treatment responses in short time (1–2 weeks)	-Advanced technology and requires special expertize	[66]
3D human brain organoids grafted with GSCs and spheres from primary and recurrent GBM	-Rapid and efficient protocol -Allow to distinguish primary and recurrent GSCs behavior -Recapitulates in vivo behavior of GSCs -Allows to study GSCs-neuron interaction -Allows adopting drug-screening assays.	-Absence of vasculatures and immune cells	[51]
4D self-transforming arrays of patient-derived organoid (PDO)	-Allows rapid drug testing (2 weeks) -Allows target and combination therapy tests	-Advanced technology and requires special expertize	[67]

*GSC* glioma stem cell, *Glioma* glioblastoma, *GBO* glioma organoid, *TME* tumor microenvironment, *PDO* patient-derived organoid, *GLICO* glioma cerebral organoids.

## Challenges in organoid-based glioma modeling

### Lack of mature neuronal and non-neuronal cell types

As described previously, stem cell-derived brain organoids is a reductionist approach containing mostly immature cell types of developing brain. Thus, these organoids perfectly fit to decode the rules that determine neural stem cell proliferation, differentiation, and primitive cortical plate formation in brain development. Glioma is a late-onset disorder, and thus more meaningful glioma modeling requires brain organoids harboring mature cell types of astrocytes, oligodendrocytes, myelinated neurons, and immune defense cell types of microglia. It is particularly important to employ organoids with a myriad of cell types because GSCs require a microenvironment. GSCs communicate, reprogram the surrounding cells, suppress the immune response, and remodel the extracellular matrix, events that all favor tumor progression. 3D tissues merely containing actively proliferating precursor cells may help obtain initial events but will not provide complex interactions occurring in glioma modeling.

To date, several protocols that generate brain organoids allow the differentiation of astrocytes, mature neuronal cell types, and even surprisingly microglia-like cells [54, 55]. Microglia are essential cell types as glioma cells communicate with them via releasing extracellular vesicles [56, 57]. Microglial cells in brain organoids are unexpected, as microglial cells do not originate from neuroectoderm, which is the primary germline to generate neural lineages. Dual-SMAD inhibition is a mechanism that can trigger the neuroectoderm formation. Ormel et al. took a thoughtful approach of generating brain organoids omitting SMAD inhibitors, which surprisingly developed Iba-1-positive microglial cells with their characteristics of ramified morphology [58]. Omitting retinoic acid at the initial stage of differentiation condition, Ramani et al. have also observed microglial cells and astrocytes in their organoids [40]. Even though it is difficult to explain the origin of non-ectodermal related cell types in directed differentiation conditions, brain organoids appear to have some degree of plasticity depending on the differentiation cues.

### Lack of endothelial cells and vasculatures

Another critical missing factor in the brain organoids is endothelial cells. It is known that GSCs reside preferentially in the vascular niches where they establish a spatial connection with endothelial cells that could affect their sensitivity to chemotherapy [59, 60]. When co-cultured with endothelial cells, brain organoids generate hybrid organoids of neuroepithelial tissue vasculatures. However, the In-Hyun Park lab devised an elegant method to generate functional vasculatures in an inducible manner by expressing ETS variant 2 that reprograms dermal fibroblasts into endothelial cells [61]. Even more surprisingly, the generated vasculature-like structures improved the organoid qualities in terms of maturation, exhibiting blood-brain barrier characteristics.

### Lack of a method that can generate organoids in large quantities

Another critical development in organoid technology is to devise a method that can generate a large number of brain organoids with minimum inter-organoid variations. In our opinion, this effort is particularly critical when organoids are used for drug screening and personalized glioma invasion assays, both of which require statistically significant data. In this direction, using a large scRNA-seq dataset, Velasco et al. demonstrated a reliable reproducibility between individual organoids that they generated [62]. Amending their approach with a method that can generate large quantities of organoids will yield a scaling-up process that can generate a large number of organoids exhibiting minimum inter-organoid variations within a batch. One of the limiting factors that cause inter-organoid variations is that the organoid generation method involves numerous steps manually, such as dispensing cells, embedding them in the matrix, and generating organoid intermediates such as embryoid bodies and neurospheres. These steps could cause aberrations, which may impact the overall homogeneity of organoids. Perhaps future semi-automated dispensing, employing pre-patterned microsphere wells and directed differentiation conditions skipping intermediate steps can help generating large numbers of brain organoids harboring disease-relevant cell types for efficient glioma modeling.

## Glioma tumoroids or GSCs?

The outcome of the glioma invasion assay depends on the starting material. As discussed above, low-passaged GSCs are accepted models of glioma [14]. However, extended passaging and altered culturing conditions can impact their heterogeneity and behavior due to selective clonal evolution in 2D cultures. An alternative to single cell GSCs is glioma tumoroids, which can be generated directly from the clinical samples by culturing them under conditions that maintain the stem cell niches. These glioma tumoroids do not differ from currently described GBOs (Glioblastoma organoids) [19]. We chose to use the term “tumoroids” since the resulting 3D objects directly emerge from clinical tumors not from self-assembling pluripotent cells. On the other hand, organoids per se mean a self-assembled 3D objects via controlled differentiation of pluripotent cells. As recently described, these glioma tumoroids mirror the histological features, complex cellular diversities, genetic signatures, and mutational profiles of their original tumor. Moreover, these glioma tumoroids can be generated quite rapidly and do not require repeated culturing in 2D. Most strikingly, when implanted in rodent brains, these glioma tumoroids exhibit aggressive infiltration in to surrounding tissues. Therefore, these 3D glioma tumoroids may be superior to GSCs in preserving the heterogeneity of parent tumor tissue. The described glioma tumoroids may contain infiltrating cells and constitute cell types that can support the infiltrating cells. Future experiments can test this aspect by conducting side-by-side comparative invasion experiments with glioma tumoroids and GSCs isolated from these tumoroids.

## Future perspectives; from simple glioma invasion assay to personalized medicine

Brain organoid models are beneficial for bridging the knowledge gap between in vitro cell cultures and in vivo animal studies. Even though GSCs are accepted models of glioma biology, GSCs may not faithfully reflect the characteristics of parent tumors. There are numerous reasons for it: (i) the culturing conditions can potentially out select a population, (ii) the artifacts of culturing conditions may suppress the heterogeneity of initial tumors, (iii) multiple passages may change the behaviors of GSCs. The glioma tumoroids can significantly address these pitfalls. When brain organoid-based glioma invasion assay is optimized with glioma tumoroids, it will be possible to scale up the reproducible assay with a reasonable turnaround time between four to six weeks after the surgery.

Besides, high-resolution imaging and computer algorithm-assisted post-processing should be in place to generate a reliable invasion map of a given clinical sample. Establishing these measures will ensure implementing the assay in a clinical setting. It is noteworthy that brain organoid invasion assays already allow distinguishing the behavior of primary and recurrent GSCs [51]. Thus, an optimized assay would enable us to stratify glioma patients based on the genetic background and the invasion behavior of patient specific GSCs. All of these developments will lay foundations to implement personalized brain organoid-based glioma invasion assays.

Brain organoid-based invasion assays hold promise for personalized drug discovery programs. Traditional treatment for glioma is the surgical resection of naïve tumors followed by radiation in combination with temozolomide (TMZ). It may be possible that organoid-based invasion assays allow us to predict the drug sensitivity of the naïve GSCs before exposure to radiation and TMZ. It is important note that drugs targeting cell cycle pathways might also affect the proliferative cells of immature organoids. To avoid this situation, one could use mature old organoids or organoid-like structures assembled from post mitotic neurons and astrocytes [63]. In the clinical setting, physicians often face glioma recurring after initial therapy. It is noteworthy that most gliomas accumulate genetic changes already at their therapy naïve status that is before the first-line treatment. Only a minor genetic change occurs in the recurrent tumor after the first-line treatment [64]. In spite of this, radio-chemotherapy is likely to affect genetic backgrounds, drug sensitivity of GSCs, and invasive behavior. For example, Goranci-Buzhala et al. described that their organoid-based glioma invasion assays had provided the first hints that recurrent GSCs are much more infiltrative in brain organoids than the GSCs derived from primary surgery [51]. It will be attractive if the described assays are scaled up to set up medium-throughput to high-throughput assays to identify agents that can mitigate the GSCs invasion. For example, identifying a battery of repurposing drugs that can impair patient derived GSCs invasion in brain organoids will greatly help therapeutics options at the clinic.

There is also a room for developing tools to conduct autologous glioma invasion assays. Currently used brain organoid-based invasion assays are performed in a homologous manner, meaning that patient-derived GSCs are grafted in different host brain organoids. Thus, another significant development is establishing autologous innovation assays by grafting patient derived GSCs into brain organoids generated from the same patient. Comparative invasion assays between homologous and autologous assays may reveal the role of diverse human brain microenvironments on tumor spread.

## Conclusion and outlook

The recently emerged 3D human brain organoids have unexpectedly offered us studying glioma biology in a new dimension, offering an excellent opportunity to visualize GSCs invasion in human brain-like tissues. With its neural stem cells, early and mature neurons, astrocytes, and microglial cells, brain organoids provide a suitable brain-like microenvironment for GSCs growth. Human brain organoids did not exist in the last decade, and organoid-based GSCs invasion assays were not there just three years ago. Hence, it is an exciting era for glioma researchers as one can map GSCs invasion in ex-vivo. However, as we discussed in the previous chapter, there are significant bottlenecks, and addressing them will allow us to model the vicious cycle of GSCs, such as the aggressive behavior of GSCs along white matter paths of the human brain (like corona radiates), peri-vascular invasion/niching and escape from immunological surveillance. From a clinical standpoint, optimizing the assays is crucial to identify responses on the sensitivity of a given tumor sample to irradiation or drug treatments within a few weeks of surgery. Such early responses will help predict patient survival after standard treatment and, hopefully, tailor personalized therapies. These innovations await future experiments.
